# Nanostructured Metal Oxide Sensors for Antibiotic Monitoring in Mineral and River Water

**DOI:** 10.3390/nano12111858

**Published:** 2022-05-29

**Authors:** Cátia Magro, Tiago Moura, Joana Dionísio, Paulo A. Ribeiro, Maria Raposo, Susana Sério

**Affiliations:** 1 School for International Training, World Learning Inc., Brattleboro, VT 05302, USA; joana.dionisio@sit.edu; 2 Department of Physics, NOVA School of Science and Technology, NOVA University Lisbon, 2829-516 Caparica, Portugal; ta.moura@campus.fct.unl.pt; 3 Laboratory of Instrumentation, Biomedical Engineering and Radiation Physics (LIBPhys-UNL), Department of Physics, NOVA School of Science and Technology, NOVA University Lisbon, 2829-516 Caparica, Portugal; pfr@fct.unl.pt (P.A.R.); mfr@fct.unl.pt (M.R.)

**Keywords:** environmental monitoring, antibiotics, impedance spectroscopy, nanostructured sensors, metal oxides, electronic tongue

## Abstract

Antibiotics represent a class of pharmaceuticals used to treat bacterial infections. However, the ever-growing use of antibiotics in agriculture and human and veterinary medicine has led to great concern regarding the outbreak of microbe strains resistant to antimicrobial drugs. Azithromycin, clarithromycin, and erythromycin are macrolides, a group of molecules with a broad spectrum of antibiotic properties, included in the second EU watchlist of emerging pollutants which emphasizes the importance of understanding their occurrence, fate, and monitoring in aquatic environments. Thus, the aim of this study was to develop sensors based on nanostructured thin films deposited on ceramic substrates with gold interdigitated electrodes, to detect azithromycin, clarithromycin, and erythromycin in water matrices (mineral and river water). Impedance spectroscopy was employed as the transducing method for the devices’ electrical signal, producing multivariate datasets which were subsequently analyzed by principal component analysis (PCA). The PCA plots for mineral water demonstrated that ZnO- and TiO_2_-based sensors produced by DC magnetron sputtering either with 50% or 100% O_2_ in the sputtering chamber, were able to detect the three macrolides in concentrations between 10^−15^ M and 10^−5^ M. In river water, the PCA discrimination presented patterns and trends, between non-doped and doped, and sorting the different concentrations of azithromycin, clarithromycin, and erythromycin. Considering both matrices, by applying the e-tongue concept, sensitivity values of 4.8 ± 0.3, 4.6 ± 0.3, and 4.5 ± 0.3 per decade to azithromycin, clarithromycin, and erythromycin concentration, respectively, were achieved. In all cases, a resolution of 1 × 10^−16^ M was found near the 10^−15^ M concentration, the lowest antibiotic concentration measured.

## 1. Introduction

The Water Research Group predicted that by 2030 more than 160% of the total available water volume in the world will be needed to satisfy global water requirements [1,2]. Water scarcity is already a reality in several countries, leading to severe economic, social, and environmental consequences, which will only be aggravated in the near future [3,4,5]. Climate change, population growth, changing consumption patterns, and the expansion of irrigated agriculture will further heighten this shortage and intensify the need to find alternatives to save freshwater [4,5,6]. Although the efficacy of wastewater treatment plants (WWTPs) has been improved in past decades, the conventional procedures applied are still not able to remove or monitor emerging pollutants (EPs); therefore, these are the main entrance vectors of these compounds in surface waters [7]. Emerging pollutants “are chemicals or microorganisms that are not commonly monitored but have the potential to enter the environment and cause adverse ecological/human health effects” [8]. Their physicochemical properties determine their persistence and bioaccumulation in the environment [7].

Water resources polluted with antibiotics increase the risk of developing antibiotic resistant bacteria and genes [9]. Azithromycin (AZI), clarithromycin (CLAR), and erythromycin (ERY) are substances with antibacterial effects for a variety of pathogenic bacteria, which have previously been detected in the final effluent of WWTPs [10,11,12]. These three macrolides were included in the latest version of the surface water watchlist, a list which proposes the need for the monitoring of 15 substances in water by EU Member States [10]. Currently, the most frequently used approach to monitor such compounds is offline monitorization, which involves sample collection followed by chemical analysis, making it difficult for real-time quantification [13]. A promising direction for environmental aqueous matrix monitoring is the application of multisensory systems [14]. Electronic tongues (e-tongues) are systems that use an array of non-specific sensors linked to data processing methods, to interpret complex responses using advanced chemometric tools in order to relate its analytical meaning [13,14]. Additionally, working electrodes in the e-tongue array can be covered with thin coatings, which highly contribute to the sensitivity and stability of the sensor’s devices. Selecting the type of film or sensorial layer are critical decisions to perform reliable qualitative and quantitative analysis [15]. Nanostructured TiO_2_ is biocompatible and non-toxic and presents a large specific surface area, exceptional electron transfer properties, and strong adsorption ability for organic and biological molecules, making it an attractive material for the development of sensor devices [16,17]. Zinc oxide is an inorganic semiconductor with strong chemical, mechanical, and thermal stability, as well as other optical and electrical properties which make it interesting for electronic, optoelectronic, and laser technology applications [18,19]. Facure et al. [20] developed an electronic tongue based on graphene hybrid nanocomposites for the detection of organophosphate (OP) pesticides in mineral and tap water matrices. The resistance data, analyzed by principal component analysis, showed the ability of these sensors to detect concentrations of OP of 0.1 nM. To test a similar thesis, in 2021, Magro et al. [21] used an array of sputtered thin films based on multi-walled carbon nanotubes and titanium dioxide, to identify triclosan concentrations ranging from 10^−15^ to 10^−5^ M in water and milk-based solutions. More recently, aligned TiO_2_ nanorod arrays decorated with closely interconnected Au/Ag nanoparticles for near-infrared surface-enhanced Raman scattering (SERS) active sensors, was used for the detection of ciprofloxacin antibiotic and chloramphenicol in environmental water samples, with detection limits of 10^–9^ M and 10^–8^ M, respectively [22].

Following the potential of those materials and the need for monitoring EP contaminants, particularly antibiotics, the aim of this study was to develop nanostructured sensors capable of discriminating concentrations in the range of 10^−15^ M to 10^−5^ M of AZI, CLAR, and ERY in mineral and river water matrices. Hence, the e-tongue concept was applied to an array of sensors composed of ceramic supports with gold interdigitated electrodes coated with thin films of titanium dioxide (TiO_2_) and zinc oxide (ZnO), deposited by DC magnetron sputtering.

## 2. Materials and Methods

All chemicals employed were of analytical grade or chemical grade (Sigma–Aldrich, St. Louis, MO, USA). The argon (Ar), oxygen (O_2_), and nitrogen (N_2_) gas, with ≥99.9% purity, were all acquired from Air Liquide (Algés, Portugal). Standards of AZI, CLAR, and ERY were purchased from Sigma-Aldrich (St. Louis, MO, USA). Ultra-pure water (resistivity of 20.4 MΩ cm at 24 °C) was obtained using a Direct-Q 3 UV system from Millipore (Bedford, MA, USA).

For this study, two experimental aqueous matrices were used: commercial Portuguese mineral water (MW) and river water (RW) collected from Tagus River at Porto Brandão, Caparica, Portugal. Table 1 presents the pH and electrical conductivity values measured for the two matrices, which were obtained using pH Prolab 1000 apparatus (Schott Instruments GmbH, Merseyside, UK).

The target compound solutions with concentrations ranging from 10^−15^ M to 10^−5^ M were prepared by performing sequential dilutions of a mother solution with a concentration of 10^−4^ M. All the solutions were prepared with an experimental matrix/MeOH (9:1). Moreover, a combination of experimental matrix/MeOH (9:1), containing no dissolved macrolides, was used as the blank standard (0 M). The samples were kept in a refrigerator unit between being prepared and their use in impedance spectroscopy measurements. The river water samples collected from Tagus River were stored in a freezing compartment.

To produce the nanostructured sensors, thin films of metal oxides were deposited on the sensorial area of ceramic substrates devices acquired from Metrohm DropSens (Oviedo, Asturias, Spain). Those were composed of two interdigitated electrodes (IDEs) with two connection tracks, all made of gold, on a ceramic substrate. The device’s dimensions were 22.8 mm (length) × 7.6 mm (width) × 1 mm (thickness), and each internal “finger” had a 200 μm width, which also corresponded to the spacing between fingers. Prior to the deposition of the thin films, all sensors were cleaned with ethanol and ultrapure water. Thereafter, the substrates were dried with compressed nitrogen gas (99% purity, Air Liquide, Algés, Portugal). Subsequently, impedance spectroscopy assays were performed on the uncoated, unused sensors exposed to surrounding air, to assure similarity between the devices.

TiO_2_ and ZnO thin films were produced by reactive DC magnetron sputtering, using titanium and zinc targets (Goodfellow, Cambridge, UK, 99.99%), respectively, as well as argon (Air Liquid, Paris, France, 99.99%) and oxygen (Gás Piedense gases, Setúbal, Portugal, 99.99%). To achieve a base pressure of 10^−4^–10^−5^ Pa (before introducing the sputtering gas) a turbomolecular pump (Pfeiffer TMH 1001, Pfeiffer Vacuum GmbH, Asslar, Germany) was utilized. Before the sputter-deposition of the films, a movable shutter was placed between the target and the supports. The target was pre-sputtered in an Ar atmosphere for 1 min to remove the target surface oxidation. The target-to-support distance was maintained at 100 mm. For each sensor device, the sputtering was performed both in a 100% O_2_ and 50:50 O_2_/Ar atmospheres and the other deposition parameters were kept constant, differing for each oxide, as summarized in Table 2.

The surface morphology was studied with a scanning electron microscope (ThermoFisher Scientific (Waltham, MA, USA) model Phenom ProX G6), operating at 15 keV. A palladium–gold thin film (~20 nm thickness) was coated on the film’s surface before SEM analysis to prevent charge build-up.

The electrical response of the sensors was assessed by measuring the impedance spectra through a Solartron 1260 Impedance/Gain-Phase Analyzer coupled to a 1296A Dielectric Interface (Solartron Analytical, AMETEK scientific instruments, Berwyn, PA, USA) by sweeping the frequency of the applied signal in the range of 1 Hz to 1 MHz. The amplitude of the stimulus was set to 25 mV due to the short spacing between the interdigitated electrodes. The parameters for the assays were processed through SMaRT Impedance Measurement Software (v. 3.3.1, AMETEK scientific instruments, Berwyn, PA, USA). Every measurement began with a 30 s delay, which allowed the system to stabilize, and was repeated twice. The impedance spectroscopy assays were performed in an ascending order of macrolide concentration. Each assay was performed in duplicate and at room temperature (~23 °C). The electrical response was replicated with a similar sensor, i.e., a sensor with a similar thin film (deposited at the same time) used as a sensitive layer, to validate the behavior of the thin films and study the reproducibility of the results measured by the nanostructured sensor devices.

The principal component analysis (PCA) was carried out considering the normalized impedance spectroscopy data (*Z*-score normalization: $z=\frac{x-\mu }{\sigma }$, with *μ* and *σ* as the mean value and standard deviation of the samples, respectively). Thus, an array of the nanostructured sensors constituted by all produced thin films was assessed as an e-tongue for AZI, CLAR, and ERY macrolides in both matrices.

## 3. Results and Discussion

### 3.1. Surface Morphology Characterization

**Figure 1:**
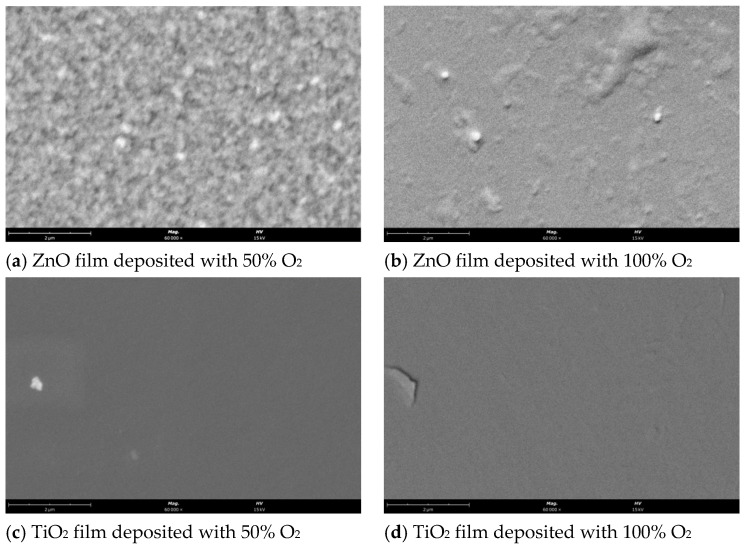

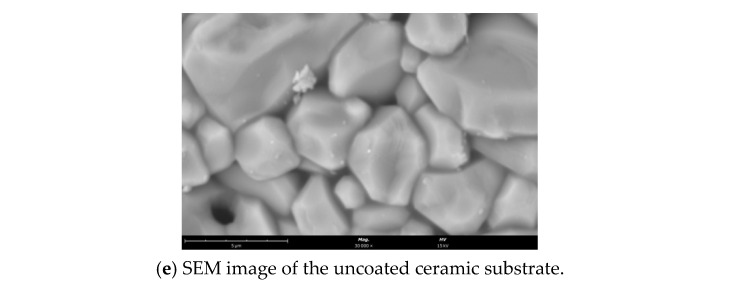
SEM images with 60,000× magnification for (**a**) ZnO 50% (60,000× magnitude); (**b**) ZnO 100%; (**c**) TiO_2_ 50%; (**d**) TiO_2_ 100% (scale bar size: 2 μm) and 30,000× magnification for (**e**) uncoated ceramic substrate (scale bar size: 5 μm).

The surface morphology of the zinc oxide and titanium dioxide thin films was analyzed by scanning electron microscopy; the obtained SEM images are depicted in Figure 1. From the SEM analysis, it can be observed that the surface of ZnO thin films is rougher than the surface of TiO_2_ films, exhibiting a granular surface microstructure, more evident for the film deposited with 50% O_2_. By increasing the oxygen percentage to 100% (i.e., only reactive gas in the chamber) the surface of the ZnO films became smoother, with fewer irregularities (Figure 1a,b; and Figure S7 in the Supplementary Materials with 30,000× magnification).

Regarding TiO_2_ (Figure 1c,d; and in Figure S7 with 30,000× magnification) both produced films exhibit similar characteristics displaying relatively uniform and smooth surface [23]. In Figure 1e, an SEM image is presented of the uncoated ceramic substrate. Although it displays quite a rough surface, it does not seem to influence the observed morphology of the thin films.

### 3.2. Impedimetric Measurements: Sputtered Thin Films

When immersed in aqueous samples, the electrical behavior of thin films deposited on the IDE is influenced by its characteristics as a sensitive layer, the properties of the double layer formed on the interface of the thin film solution, and the attributes of the bulk electrolyte [24]. Moreover, the electrical behavior is analogous to an equivalent circuit with components that represent the thin film, double layer, and electrolyte; thus, it may be interpreted by the electrical properties of that circuit [25]. Prior to the analysis of the results, the reproducibility was evaluated. Thus, it may be observed by the Tables S1–S4 in the Supplementary Materials that higher values of standard deviation could be explained by the slight heterogeneity which may be experienced upon the sputtering of the thin films onto the substrate. Although the pairs of films were deposited at the same time, the substrates’ positions, regarding the magnetron cathode, may differ slightly; therefore, this may lead to very small discrepancies in the thickness of the films that justify the observed standard deviation. Nonetheless, the results were consistent and reproducible, presenting the same trends on the curves for impedance data. Accordingly, the impedance spectra measured by the different thin film sensors immersed in MW and RW samples of the three macrolides are presented in the subsequent sections.

#### 3.2.1. ZnO Thin Films Deposited with 50% and 100% O_2_

**Figure 2:**
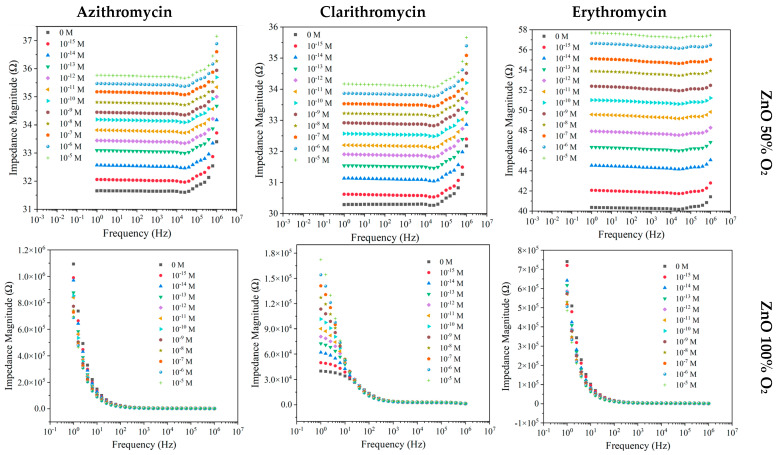
Impedance spectra of the ZnO sensor devices deposited with 50% and 100% O_2_ measured at different frequencies, when immersed in MW at different AZI, CLA, and ERY concentrations.

In Figure 2, the response of ZnO films produced with 50% and 100% of O_2_ is presented, with respect to the different antibiotics in MW.

In the low-conductivity matrix, the sensor coated with the ZnO thin film deposited with 50% O_2_ showed a clear pattern of increasing impedance with the increases in AZI, CLAR, and ERY concentrations in the entire frequency range studied (Figure 2). Nonetheless, for ERY, the range of sensitivity is more evident in higher-frequency values. As shown in Figure 2, the electrical measurements obtained with the ZnO sensors produced with 100% O_2_ exhibited a similar behavior for all antibiotics, showing a more pronounced sensitivity between 1 and 10 Hz in CLAR samples. By fixing the impedance in relation to compound concentrations, the devices that presented larger sensitivity for CLARY and ERY were ZnO sensors produced with 100% O_2_ and 50% O_2_, respectively (Figures S3 and S5). Those sensors exhibited identical trends of monotonic functions with inverse tendency.

**Figure 3:**
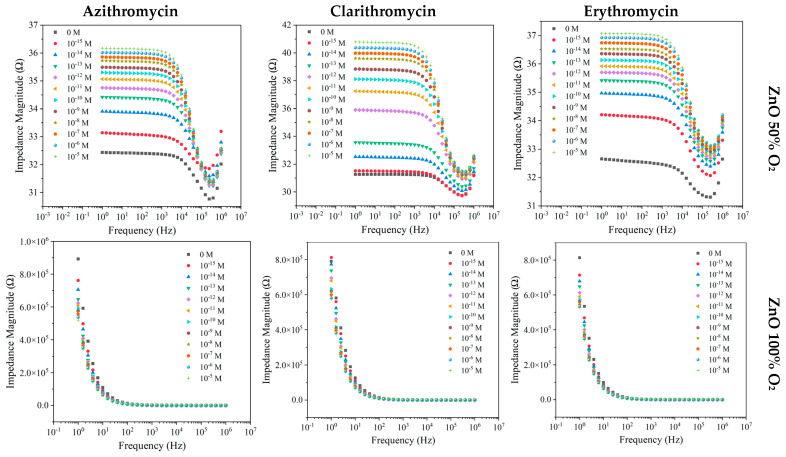
Impedance spectra of the ZnO sensors devices deposited with 50% and 100% O_2_ measured at different frequencies, when immersed in RW at different AZI, CLAR, and ERY concentrations.

Measuring the target compounds in river water means the change in the conductivity of the media and its pH; therefore, this may influence the impedance behavior (Figure 3).

Changing the matrix did not show variation in the trend of the impedance, which increased with the increase in concentration. Nevertheless, for all antibiotics, a more pronounced sensitivity in the 1–10 Hz frequency range (Figure 3) is observed, due to the impedimetric curve change at 10,000 Hz. Additionally, the fluctuation of the impedance values achieved for ZnO sensors deposited with 50% O_2_ narrowed from 10^−5^ M to 10^−12^ M, as compared with the amplitude from 0 M to 10^−12^ M For ZnO (50% O_2_), by fixing the impedance at 6.31 × 10^4^ and 3.98 × 10^2^ Hz in relation to compound concentrations, resulted in an increasing normalized impedance trend for both AZI and CLAR (Figures S2 and S4). Regarding ERY, the ZnO sensor produced with 50% of O_2_ presented a normalized impedance increase between 10^−15^ and 10^−8^ M; however, the device reached saturation point, which can be seen by the approximately constant impedance tendency (Figure S6). In the case of the ZnO (100% O_2_) sensor, when the frequency was fixed at 1.58 Hz for ERY and 1 Hz for both AZI and CLAR (Figures S2, S4 and S6), all the compounds under study displayed a decreasing pattern, proving that this sensor exhibits higher sensitivity in the studied concentration range.

#### 3.2.2. TiO_2_ Thin Films Produced with 50% O_2_ and 100% O_2_

**Figure 4:**
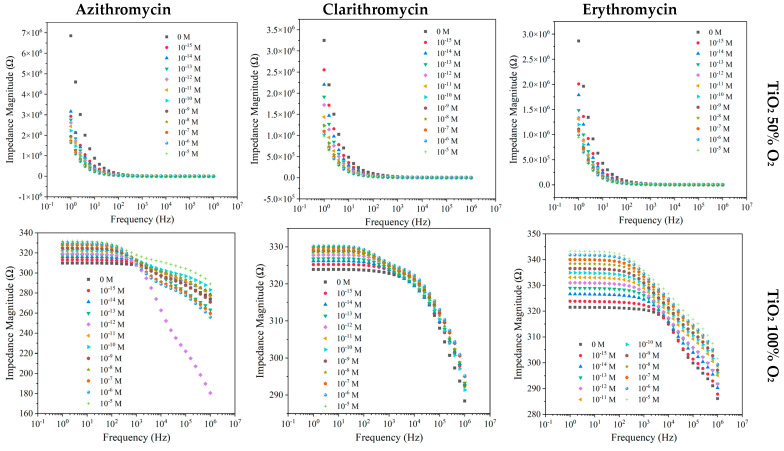
Impedance spectra of the TiO_2_-based sensors device produced with 50% and 100% O_2_ measured at different frequencies, when immersed in MW at different AZI, CLAR, and ERY concentrations.

The electrical responses of TiO_2_ films produced with 50% and 100% of O_2_ to the different antibiotics in MW matrices are depicted in Figure 4.

When the considered matrix was MW, similar footprints regarding AZI, CLAR, and ERY were observed for the TiO_2_ (50% O_2_) sensor, with the measured impedance magnitude presenting significant sensitivity at lower frequencies (1–100 Hz) with a decreasing trend (Figure 4). The TiO_2_ sensor produced with 100% O_2_ exhibited increased impedance with the increases in AZI, CLAR, and ERY concentrations, displaying substantial sensitivity in the frequency range of 1 Hz to 1 kHz, more pronounced in the case of ERY samples (Figure 4). The impedance measured for TiO_2_ thin film sensors was normalized in relation to the blank concentration (Figure S1). The results achieved with AZI samples show how the sensors with TiO_2_ acted in contrast to one another, with TiO_2_ (100% O_2_) thin films showing an increasing impedance trend and TiO_2_ (50% O_2_) presenting a decreasing impedance trend. Both types of sensors presented significant sensitivity to the distinct AZI concentrations. A decreasing trend was also observed in TiO_2_ (50% O_2_) for CLAR until the concentration of 10^−6^ M, where it seemed to have reached a sensor saturation (Figure S3). Likewise, this sensor saturation was observed for the ERY compound above 10^−7^ M (Figure S5). For CLAR and ERY, the TiO_2_ (100% O_2_) sensor exhibited a dispersion in results, evidencing a lower sensitivity to ERY.

**Figure 5:**
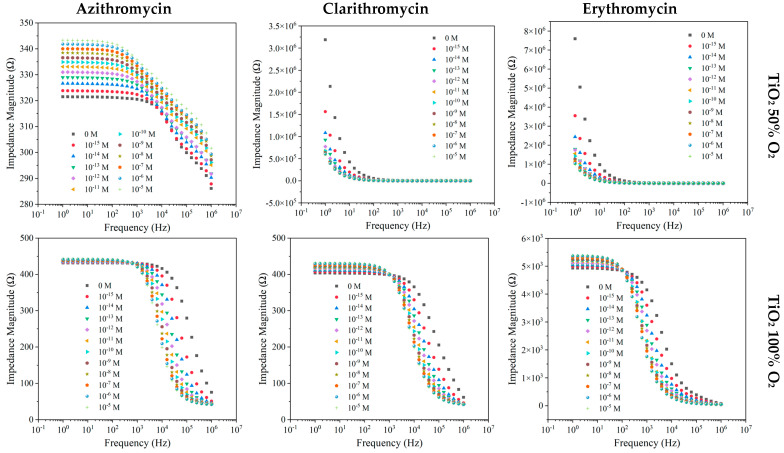
Impedance spectra at different frequencies of the TiO_2_-based sensor devices produced with 50% and 100% O_2_, when immersed in RW at different AZI, CLA, and ERY concentrations.

Impedance spectra of RW samples with AZI, CLAR, or ERY show similar patterns to those observed in the MW samples for a TiO_2_ sensor produced with 50% O_2_, displaying a more pronounced sensitivity in the 1 Hz to 10 Hz frequency range (Figure 5).

Concerning the TiO_2_ sensor produced with 100% O_2_, the device showed sensitivity to two distinct frequency ranges, for AZI, CLAR, and ERY. The impedance values increased between 1 Hz and 100 Hz and 1 Hz and 1 kHz for AZI and CLAR, respectively. Above 1 MHz for AZI and 1 kHz for CLAR, a decreasing impedance trend was observed. As previously mentioned, by normalizing the impedance values to a fixed frequency of 1 Hz for AZI and ERY and 10^6^ Hz for CLAR (Figures S2, S4 and S6), it could be observed that the results achieved with the TiO_2_ device produced with 50% O_2_ only showed sensitivity for AZI and ERY samples between 10^−15^ and 10^−8^ M. On the other hand, for CLAR samples, the TiO_2_ (100% O_2_) sensor displayed a more pronounced sensitivity in comparison with the TiO_2_ (50% O_2_) sensor. Finally, the function that fit the data measured by the TiO_2_ (100% O_2_) follows an impedance increasing trend.

### 3.3. Electronic Tongue Concept

#### Principal Component Analysis: Sensor Sensitivity and Resolution

**Figure 6:**
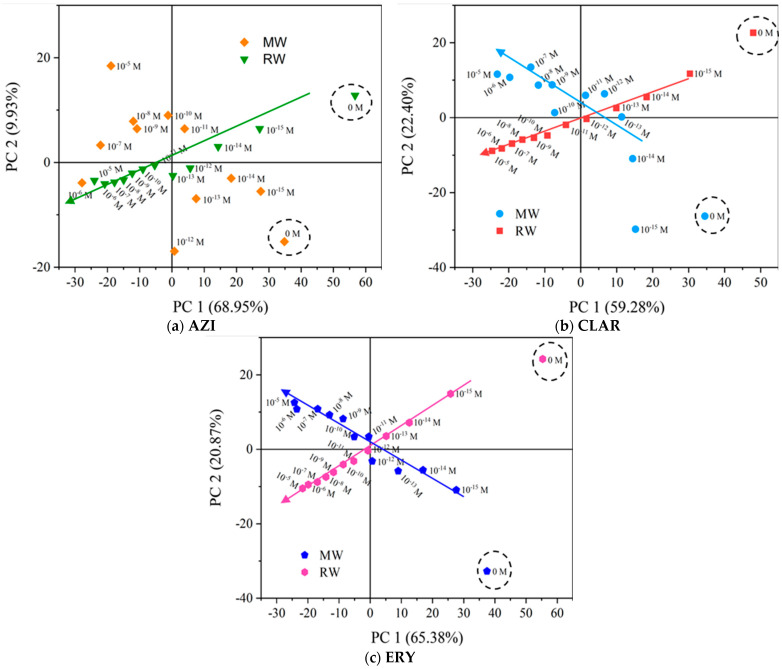
PCA score plot for the electronic tongue concept for AZI (**a**), CLAR (**b**), and ERY (**c**) in ranges of concentration from 0 M to 10^−15^ M to 10^−5^ M, when immersed in MW and RW.

Following the analysis of the electrical response and capabilities of the individual nanostructured sensors, the electronic tongue concept was assessed through PCA, and applied to the impedance, capacitance, resistance, reactance, and loss tangent dataset of an array of sensors composed by all the thin film sensors produced in the present study. The e-tongue sensor was used to distinguish macrolide concentrations in both experimental matrices. The resulting PCA plots are presented in Figure 6.

Overall, the first two principal components, PC 1 and PC 2, accounted for above 79% of the total variance in the three electronic tongues. From Figure 6a, a clear decreasing trend in PC 1 with respect to AZI concentration in RW was observed. In contrast, although the PCA plot discriminated the different AZI concentrations in MW, no trends or patterns were observed. Regarding the e-tongue for CLAR, the PCA plot of the e-tongue sensor (Figure 6b) very clearly distinguishes different concentrations in RW across the main axis, PC 1. Within MW, a pattern of PC 1 is also observed. In both matrices, 0 M is in the equivalent quadrant of the lowest concentration, which stresses the ability to distinguish different concentrations of CLAR. Figure 6c shows a clear trend along the main axis, similar to, although more pronounced when compared with the AZI and CLAR PCA plots. Lastly, the three PCA plots showed that the e-tongue device could discriminate between non-doped samples and samples spiked with AZI, CLAR, and ERY, regardless of the matrix.

Considering the trends and patterns found in the PCA plots (Figure 6) of the data measured by the various thin film sensors, the principal component (PC1) that presented the most notable and significant tendency with respect to the antibiotics’ concentration, in each case, was plotted as a function of concentration. Thus, because it was observed as a linear trend of the principal component factor score, it was possible to use the PC1 data to determine the sensors’ sensitivity for the three macrolides and their resolution.

The sensitivity corresponded to the slope of the linear function that adequately fitted the plotted data. The resolution was found near the smallest concentration of the implicit linear range (*C_S_*), through the following equation, considering the minimum value that could be measured and the error associated with the sensitivity:

ΔlogC=logC−logCs

where $\Delta logC=\frac{error}{sensitivity}$; therefore, the resolution is equal to C−Cs.

**Figure 7:**
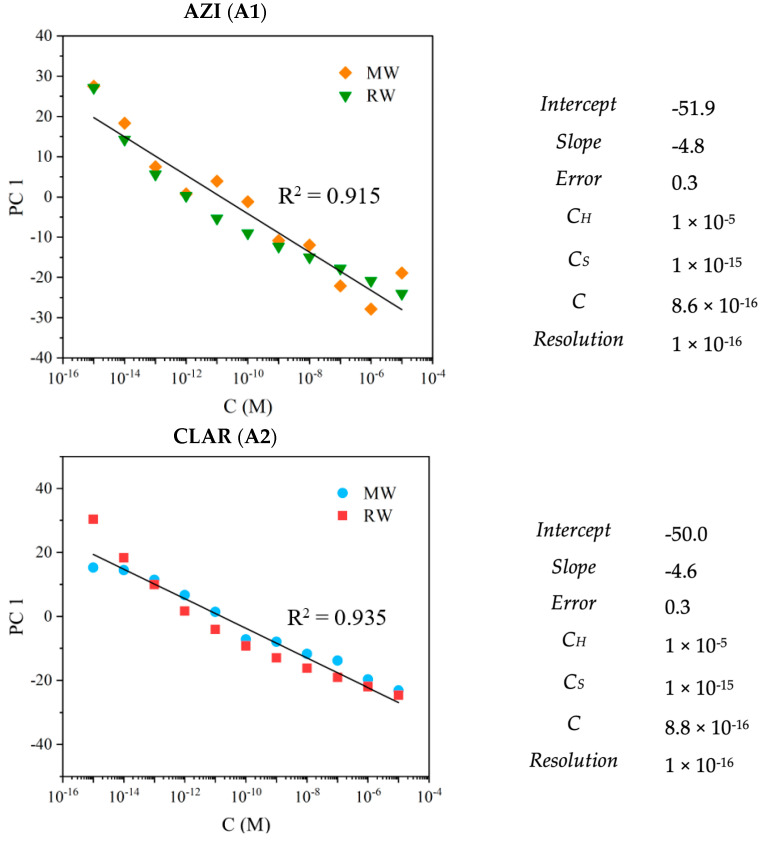

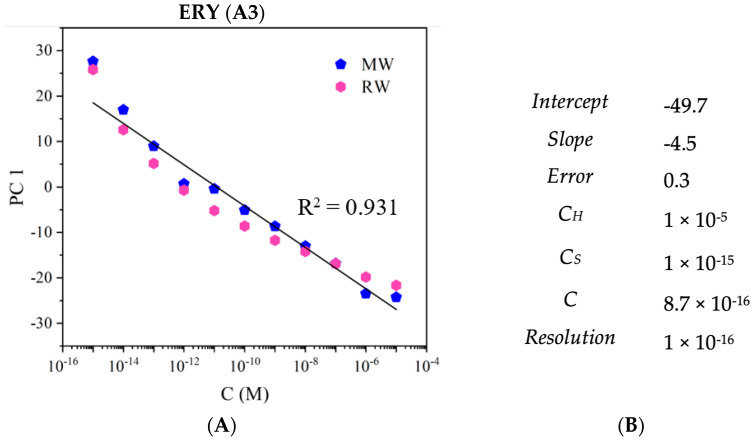
(**A**) PC1 factor scores as a function of AZI (**A1**), CLAR (**A2**), and ERY (**A3**) concentrations (0 M to 10^−15^ M to 10^−5^ M), distinguished with the electronic tongue build-up with TiO_2_- and ZnO-based sensors, when immersed in MW and RW; (**B**) linear range, sensitivity, and resolution for each macrolide.

The factor scores of PC 1 obtained for both matrices as a function of AZI, CLAR, and ERY concentration and its operational parameters (linear range information, sensitivity, and resolution) are presented in Figure 7. As can be observed in Figure 7, the sensor array chosen in this paper showed similar sensitivity and equal resolution for all the macrolides under study.

**Figure 8:**
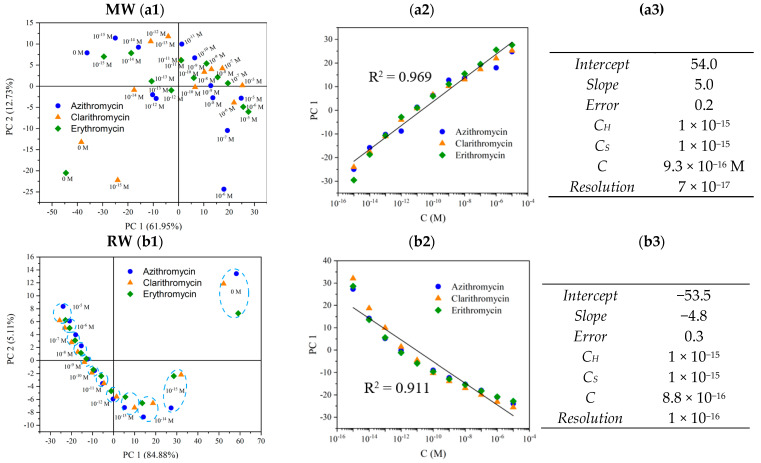
Principal component analysis (PCA) score plot for the electronic tongue concept for AZI, CLA, and ERY in the range of concentrations from 0 M to 10^−15^ M to 10^−5^ M, when immersed in MW (**a1**) and RW (**b1**); PC1 factor scores as a function of AZI, CLAR, and ERY concentrations (0 M to 10^−15^ M to 10^−5^ M) when immersed in MW (**a2**) and RW (**b2**); linear range, sensitivity, and resolution for each macrolide in MW (**a3**) and RW (**b3**).

Moreover, a PCA plot was constructed to understand the capability of the proposed e-tongue to distinguish between the three antibiotics in the two matrices (Figure 8).

The observations depicted in Figure 8 are in line with previous results [15,24], which have highlighted the essential role of ionic media and pH–pKa relationships in compound adsorption or non-absorption onto thin films. In the present study, the target compounds were in non-ionized form in mineral water; in river water they could be found in both forms (ionized and non-ionized). Thus, there are two outlines in both Figure 8a,b. In Figure 8(a1), although the ranges of concentrations are typically in the same quadrant, namely in the second and third areas, no trend is observed. In contrast, in Figure 8(b1), there is a clear trend and pattern between the detection of the target compounds. Nevertheless, with those two e-tongues it was possible to attain a device with sensitivity of 5.0 ± 0.2 and −4.8 ± 0.3 per decade in MW and RW spiked with AZI, CLAR, and ERY. For both e-tongues, resolutions of 7.0 × 10^−17^ M and 1 × 10^−16^ M were achieved. The proposed e-tongue presents a lower detection limit when compared with other promising sensors developed for the monitoring of antibiotics and/or organic compounds: (1) In 2013, Zhang et al. developed multi-walled carbon nanotubes (MWCNTs) for azithromycin detection with a detection limit in a water sample of 1.1 × 10^−9^–6 × 10^−9^ M [26]; Khanna et al. (2018) tested a MnO_2_ electrochemical sensor to detect BPA and achieved a detection limit of 0.66 μM [27]; in 2020, Ayankojo et al. derived a molecularly imprinted polymer with a portable electrochemical transducer, a screen-printed electrode, and could detect erythromycin in tap water at concentrations lower than 0.1 nM [28]. Thus, reflecting upon the data observed in Figure 8, the superior ability of the e-tongue to present patterns and trends in higher pH and conductivity matrices is clear, as was the case in RW. Finally, it is evident that the analogous characteristics and morphology may explain the data observed in a matrix where there are 50% of ionized molecules. This confirms that these types of sensors, working as an e-tongue, may be considered as a measuring technique; therefore, they could be further used to develop an optimized antibiotic device working as a monitoring tool on aqueous matrices with different complexities.

## 4. Conclusions

The development of an array of nanostructured TiO_2_ and ZnO sensors for the detection of AZI, CLAR, and ERY in concentrations between 10^−15^ M and 10^−5^ M in MW and RW matrices has been proposed. Impedance spectra measured by sensors coated with ZnO and TiO_2_ thin films deposited with 50% and 100% O_2_ by DC magnetron sputtering showed different footprints according to the experimental matrix. However, the electrical behaviors of the different thin film sensors were identical for the three macrolides. Overall, the nanostructured ZnO and TiO_2_ sensors produced impedance spectra with distinct trends and patterns regarding macrolide concentrations.

Normalized impedance, measured at fixed frequencies by the different devices as a function of concentration, was used to determine monotone functions of macrolide concentrations that allowed operational measurements of the analytes. The ZnO thin film showed a better ability to work as a detection sensor for the target compounds in comparison with TiO_2_. This may be due to its higher roughness and surface area that allowed the adsorption of the compound and further detection with impedance systems. Additionally, ZnO seemed to present more robustness to pH fluctuations and matrix characteristics (good results in both matrices), as well as greater sensitivity to a higher range of concentrations and frequencies.

Additionally, the electronic tongue concept was applied to the full range of sensors under study. The e-tongue sensor’s PCA plots indicated that the proposed device could discriminate between the two experimental matrices. The e-tongue was further capable of detecting AZI, CLAR, and ERY in both matrices, and quantify the three macrolides in RW in the complete concentration range. Thus, by applying the e-tongue concept, sensitivities of 4.8 ± 0.3, 4.6 ± 0.3, and 4.5 ± 0.3 per decade to AZI, CLAR, and ERY concentration were achieved, respectively. In all cases, a resolution of 1 × 10^−16^ M was found near the 10^−15^ M concentration, the smallest concentration that could be quantified. Lastly, it was revealed that the proposed e-tongue, with a sensitivity of −4.8 ± 0.3 per decade and a resolution of 1 × 10^−16^ M, could recognize the different ranges of concentrations with a clear trend, but not a specific molecule in river water.

## Figures and Tables

**Table 1:** pH and electrical conductivity values measured for mineral and river water.

	Mineral Water	River Water
pH	5.875 ± 0.001	7.536 ± 0.001
Electrical Conductivity (mS/cm)	11.62 ± 0.01	52.43 ± 0.01

**Table 2:** Sputtering thin film production characteristics.

	ZnO_50	ZnO_100	TiO_2__50	TiO_2__100
O_2_ (%)	50%	100%	50%	100%
Ar (%)	50%	0%	50%	0%
Working Pressure (Pa)	0.8	0.8	0.8	0.8
Power (W)	300	290	1010	1020
Voltage (V)	462	275	302	376
Current (A)	0.65	1.04	3.34	2.70
Time (min)	5	5	15	15

## Data Availability

Not applicable.

## Supplementary Materials

The following supporting information can be downloaded at: https://www.mdpi.com/article/10.3390/nano12111858/s1, Figure S1. Normalized impedance as a function of azithromycin concentration in MW at fixed frequencies: 1, 1.58, 2.51, and 2.51 × 10^3^ Hz measured by the TiO_2_ (100% O_2_) sensor, TiO_2_ (50% O_2_) sensor, ZnO (100% O_2_) sensor, and ZnO (50% O_2_) sensor, respectively; Figure S2. Normalized impedance as a function of azithromycin concentration in RW at fixed frequencies: 1, 1.58, 3.98 × 10^3^, and 6.31 × 10^4^ Hz measured by the TiO_2_ (50% O_2_) sensor, ZnO (100% O_2_) sensor, TiO_2_ (100% O_2_) sensor, and ZnO (50% O_2_) sensor, respectively; Figure S3. Normalized impedance as a function of clarithromycin concentration in MW at fixed frequencies: 1, 1, 2.51, and 2.51 Hz measured by the TiO_2_ (50% O_2_) sensor, TiO_2_ (100% O_2_) sensor, ZnO (50% O_2_) sensor, and ZnO (100% O_2_) sensor, respectively; Figure S4. Normalized impedance as a function of clarithromycin concentration in RW at fixed frequencies: 1, 3.98 × 102, 2.51 × 10^3^ and 10^6^ Hz measured by the ZnO (100% O_2_) sensor, ZnO (50% O_2_) sensor, TiO_2_ (100% O_2_) sensor, and TiO_2_ (50% O_2_) sensor, respectively; Figure S5. Normalized impedance as a function of erythromycin concentration in MW at fixed frequencies: 1, 1.58 × 10^2^ and 2.51 × 10^3^ Hz measured by the TiO_2_ (50% O_2_) sensor, ZnO (50% O_2_) sensor, ZnO (100% O_2_), and TiO_2_ (100% O_2_) sensor, respectively; Figure S6. Normalized impedance as a function of erythromycin concentration in RW at fixed frequencies: 1, 10, 2.51 × 10^4^, and 10^6^ Hz measured by the TiO_2_ (50% O_2_) sensor, TiO_2_ (100% O_2_) sensor, ZnO (50%O_2_) sensor, and ZnO (100% O_2_) sensor, respectively. Figure S7. SEM images with 30,000 times magnification for (a) ZnO 50%; (b) ZnO 100%; (c) TiO_2_ 50%; (d) TiO_2_ 100%. Table S1. Normalized Impedance data (Ω) reproducibility of the TiO_2_ based sensors devices produced with 100% O_2_, when immersed in MW and RW at different AZI, CLA and ERY concentrations. Table S2. Normalized Impedance data (Ω) reproducibility of the TiO_2_ based sensors devices produced with 50% O_2_, when immersed in MW and RW at different AZI, CLA and ERY concentrations. Table S3. Normalized Impedance data (Ω) reproducibility of the ZnO based sensors devices produced with 100% O_2_, when immersed in MW and RW at different AZI, CLA and ERY concentrations. Table S4. Normalized Impedance data (Ω) reproducibility of the ZnO based sensors devices produced with 50% O_2_, when immersed in MW and RW at different AZI, CLA and ERY concentrations.
