# Supplementary material for: Nanostructured Metal Oxide Sensors for Antibiotic Monitoring in Mineral and River Water
Source: Nanomaterials (Basel). 2022 May 29;12(11):1858. doi: 10.3390/nano12111858 (PMC9182526; doi:10.3390/nano12111858)
Supplement: Supplementary file 1 [file nanomaterials-12-01858-s001.zip › nanomaterials-1736061-supplementary.pdf]

Supplementary Data

Nanostructured Metal Oxide Sensors for Antibiotic Monitoring in Mineral and River Water

Cátia Magro [1,2,*], Tiago Moura [2], Joana Dionísio [1], Paulo A. Ribeiro [3], Maria Raposo [3] and Susana Sério [3,*]

[1] School for International Training, World Learning Inc., Brattleboro, VT 05302, USA; joana.dionisio@sit.edu
[2] Department of Physics, NOVA School of Science and Technology, NOVA University Lisbon, 2829-516 Caparica, Portugal; ta.moura@campus.fct.unl.pt
[3] Laboratory of Instrumentation, Biomedical Engineering and Radiation Physics (LIBPhys-UNL), Department of Physics, NOVA School of Science and Technology, NOVA University Lisbon, 2829-516 Caparica, Portugal; pfr@fct.unl.pt (P.A.R.); mfr@fct.unl.pt (M.R.)
* Correspondence: catia.magro@sit.edu (C.M.); susana.serio@fct.unl.pt (S.S.)

S1. Reproducibility Measurements of Figures S1–S6.

Table S1. Normalized Impedance data (Ω) reproducibility of the TiO_2 based sensors devices produced with 100% O_2, when immersed in MW and RW at different AZI, CLA and ERY concentrations.

MW AZI				
Concentrations	Sensor 1	Sensor 2	Average	Standard Deviation
10^{-15} M	0.0106	0.0039	0.0072	0.0034
10^{-14} M	0.0200	0.0073	0.0137	0.0063
10^{-13} M	0.0254	0.0094	0.0174	0.0080
10^{-12} M	0.0300	0.0117	0.0208	0.0091
10^{-11} M	0.0390	0.0150	0.0270	0.0120
10^{-10} M	0.0432	0.0173	0.0303	0.0129
10^{-9} M	0.0484	0.0187	0.0336	0.0148
10^{-8} M	0.0547	0.0207	0.0377	0.0170

MW CLARI				
Concentrations	Sensor 1	Sensor 2	Average	Standard Deviation
10^{-15} M	0.0041	0.0056	0.0048	0.0008
10^{-14} M	0.0070	0.0106	0.0088	0.0018
10^{-13} M	0.0091	0.0133	0.0112	0.0021
10^{-12} M	0.0118	0.0155	0.0137	0.0018
10^{-11} M	0.0139	0.0234	0.0187	0.0048
10^{-10} M	0.0152	0.0285	0.0219	0.0067
10^{-9} M	0.0158	0.0213	0.0185	0.0028
10^{-8} M	0.0159	0.0230	0.0194	0.0035

MW ERIT				
Concentrations	Sensor 1	Sensor 2	Average	Standard Deviation
10^{-15} M	−0.007121814	0.003715304	−0.0017	0.0054
10^{-14} M	−0.010836905	0.008461669	−0.0012	0.0096
10^{-13} M	−0.014426082	0.011704099	−0.0014	0.0131
10^{-12} M	−0.017404262	0.013934241	−0.0017	0.0157
10^{-11} M	−0.017649471	0.018165389	0.0003	0.0179
10^{-10} M	−0.017293276	0.021319272	0.0020	0.0193
10^{-9} M	−0.02298693	0.023543261	0.0003	0.0233
10^{-8} M	−0.020006048	0.02746048	0.0037	0.0237

10^{-7} M	0.0591	0.0218	0.0404	0.0187
10^{-6} M	0.0643	0.0244	0.0443	0.0200
10^{-5} M	0.0706	0.0271	0.0489	0.0217

10^{-7} M	0.0181	0.0546	0.0364	0.0182
10^{-6} M	0.0194	0.0746	0.0470	0.0276
10^{-5} M	0.0192	0.0321	0.0256	0.0065

10^{-7} M	−0.021724946	0.030946648	0.0046	0.0263
10^{-6} M	−0.021508108	0.03403566	0.0063	0.0278
10^{-5} M	−0.01754882	0.039208488	0.0108	0.0284

RW AZI				
Concentrations	Sensor 1	Sensor 2	Average	Standard Deviation
10^{-15} M	−0.0126	−0.0007	−0.0066	0.0060
10^{-14} M	−0.0306	−0.0023	−0.0165	0.0141
10^{-13} M	−0.0529	−0.0029	−0.0279	0.0250
10^{-12} M	−0.0773	−0.0040	−0.0406	0.0367
10^{-11} M	−0.0971	−0.0048	−0.0510	0.0462
10^{-10} M	−0.1205	−0.0066	−0.0635	0.0570
10^{-9} M	−0.1480	−0.0092	−0.0786	0.0694
10^{-8} M	−0.1767	−0.0098	−0.0932	0.0835
10^{-7} M	−0.2013	−0.0116	−0.1065	0.0948
10^{-6} M	−0.2252	−0.0150	−0.1201	0.1051
10^{-5} M	−0.2454	−0.0155	−0.1305	0.1150

RW CLARI				
Concentrations	Sensor 1	Sensor 2	Average	Standard Deviation
10^{-15} M	−0.0383	−0.0160	−0.0272	0.0112
10^{-14} M	−0.0576	−0.0276	−0.0426	0.0150
10^{-13} M	−0.0732	−0.0371	−0.0551	0.0180
10^{-12} M	−0.0866	−0.0455	−0.0661	0.0206
10^{-11} M	−0.1125	−0.0588	−0.0857	0.0269
10^{-10} M	−0.1251	−0.0673	−0.0962	0.0289
10^{-9} M	−0.1415	−0.0764	−0.1090	0.0326
10^{-8} M	−0.1581	−0.0862	−0.1221	0.0360
10^{-7} M	−0.1784	−0.0956	−0.1370	0.0414
10^{-6} M	−0.1943	−0.1100	−0.1521	0.0422
10^{-5} M	−0.2144	−0.1140	−0.1642	0.0502

RW ERIT				
Concentrations	Sensor 1	Sensor 2	Average	Standard Deviation
10^{-15} M	0.0041	0.0120	0.0080	0.0039
10^{-14} M	0.0043	0.0204	0.0123	0.0081
10^{-13} M	0.0094	0.0277	0.0186	0.0091
10^{-12} M	0.0107	0.0356	0.0231	0.0124
10^{-11} M	0.0142	0.0477	0.0310	0.0168
10^{-10} M	0.0180	0.0547	0.0363	0.0183
10^{-9} M	0.0212	0.0609	0.0411	0.0198
10^{-8} M	0.0234	0.0698	0.0466	0.0232
10^{-7} M	0.0274	0.0767	0.0520	0.0246
10^{-6} M	0.0308	0.0846	0.0577	0.0269
10^{-5} M	0.0325	0.0887	0.0606	0.0281

Table S2. Normalized Impedance data (Ω) reproducibility of the TiO_2 based sensors devices produced with 50% O_2, when immersed in MW and RW at different AZI, CLA and ERY concentrations.

MW AZI				
Concentrations	Sensor 1	Sensor 2	Average	Standard Deviation
10^{-15} M	−0.5355	−0.2953	−0.4154	0.1201
10^{-14} M	−0.5394	−0.3480	−0.4437	0.0957
10^{-13} M	−0.5986	−0.4575	−0.5280	0.0705
10^{-12} M	−0.6275	−0.4443	−0.5359	0.0916

MW CLARI				
Concentrations	Sensor 1	Sensor 2	Average	Standard Deviation
10^{-15} M	−0.3162	−0.2142	−0.2652	0.0510
10^{-14} M	−0.3885	−0.3214	−0.3550	0.0336
10^{-13} M	−0.4492	−0.4107	−0.4299	0.0193
10^{-12} M	−0.4677	−0.4700	−0.4688	0.0012

MW ERIT				
Concentrations	Sensor 1	Sensor 2	Average	Standard Deviation
10^{-15} M	−0.298638445	−0.289563448	−0.2941	0.0045
10^{-14} M	−0.375538138	−0.388109988	−0.3818	0.0063
10^{-13} M	−0.481711642	−0.452344157	−0.4670	0.0147
10^{-12} M	−0.533672326	−0.49143296	−0.5126	0.0211

10^{-11} M	−0.6473	−0.4753	−0.5613	0.0860
10^{-10} M	−0.6793	−0.5020	−0.5907	0.0887
10^{-9} M	−0.7241	−0.5479	−0.6360	0.0881
10^{-8} M	−0.7270	−0.5337	−0.6304	0.0967
10^{-7} M	−0.7520	−0.5442	−0.6481	0.1039
10^{-6} M	−0.7631	−0.5455	−0.6543	0.1088
10^{-5} M	−0.7644	−0.5480	−0.6562	0.1082

10^{-11} M	−0.4981	−0.5585	−0.5283	0.0302
10^{-10} M	−0.5452	−0.6201	−0.5827	0.0375
10^{-9} M	−0.5430	−0.6624	−0.6027	0.0597
10^{-8} M	−0.5660	−0.6192	−0.5926	0.0266
10^{-7} M	−0.6192	−0.6621	−0.6406	0.0215
10^{-6} M	−0.5851	−0.6835	−0.6343	0.0492
10^{-5} M	−0.6135	−0.7013	−0.6574	0.0439

10^{-11} M	−0.543996559	−0.514074815	−0.5290	0.0150
10^{-10} M	−0.580831031	−0.526168915	−0.5535	0.0273
10^{-9} M	−0.612072783	−0.55132712	−0.5817	0.0304
10^{-8} M	−0.620737614	−0.566044259	−0.5934	0.0273
10^{-7} M	−0.62728041	−0.567301508	−0.5973	0.0300
10^{-6} M	−0.653309718	−0.604771693	−0.6290	0.0243
10^{-5} M	−0.669442648	−0.586878472	−0.6282	0.0413

RW AZI				
Concentrations	Sensor 1	Sensor 2	Average	Standard Deviation
10^{-15} M	−0.5100	−0.4319	−0.4709	0.0390
10^{-14} M	−0.6604	−0.4829	−0.5716	0.0887
10^{-13} M	−0.7097	−0.5731	−0.6414	0.0683
10^{-12} M	−0.7579	−0.6480	−0.7030	0.0550
10^{-11} M	−0.7888	−0.6509	−0.7199	0.0690
10^{-10} M	−0.7884	−0.6783	−0.7334	0.0550
10^{-9} M	−0.8004	−0.6726	−0.7365	0.0639
10^{-8} M	−0.8049	−0.6930	−0.7490	0.0560
10^{-7} M	−0.8108	−0.6932	−0.7520	0.0588
10^{-6} M	−0.8103	−0.7073	−0.7588	0.0515
10^{-5} M	−0.8150	−0.7093	−0.7622	0.0529

RW CLARI				
Concentrations	Sensor 1	Sensor 2	Average	Standard Deviation
10^{-15} M	−0.0441	−0.0396	−0.0419	0.0023
10^{-14} M	−0.0575	−0.0480	−0.0528	0.0048
10^{-13} M	−0.0603	−0.0531	−0.0567	0.0036
10^{-12} M	−0.0589	−0.0568	−0.0578	0.0010
10^{-11} M	−0.0590	−0.0562	−0.0576	0.0014
10^{-10} M	−0.0598	−0.0566	−0.0582	0.0016
10^{-9} M	−0.0632	−0.0595	−0.0614	0.0018
10^{-8} M	−0.0642	−0.0596	−0.0619	0.0023
10^{-7} M	−0.0658	−0.0603	−0.0630	0.0027
10^{-6} M	−0.0675	−0.0613	−0.0644	0.0031
10^{-5} M	−0.0689	−0.0603	−0.0646	0.0043

RW ERIT				
Concentrations	Sensor 1	Sensor 2	Average	Standard Deviation
10^{-15} M	−0.4258	−0.4472	−0.4365	0.0107
10^{-14} M	−0.6010	−0.5913	−0.5962	0.0048
10^{-13} M	−0.6495	−0.7111	−0.6803	0.0308
10^{-12} M	−0.6773	−0.7205	−0.6989	0.0216
10^{-11} M	−0.6977	−0.7394	−0.7186	0.0208
10^{-10} M	−0.7129	−0.7539	−0.7334	0.0205
10^{-9} M	−0.7255	−0.7654	−0.7455	0.0200
10^{-8} M	−0.7361	−0.7697	−0.7529	0.0168
10^{-7} M	−0.7415	−0.7731	−0.7573	0.0158
10^{-6} M	−0.7577	−0.7777	−0.7677	0.0100
10^{-5} M	−0.7615	−0.7819	−0.7717	0.0102

Table S3. Normalized Impedance data (Ω) reproducibility of the ZnO based sensors devices produced with 100% O_2, when immersed in MW and RW at different AZI, CLA and ERY concentrations.

MW AZI				
Concentrations	Sensor 1	Sensor 2	Average	Standard Deviation
10^{-15} M	−0.0932	0.3406	0.1237	0.2169
10^{-14} M	−0.1248	0.4380	0.1566	0.2814
10^{-13} M	−0.2085	0.4313	0.1114	0.3199
10^{-12} M	−0.2404	0.3799	0.0697	0.3101
10^{-11} M	−0.2473	0.3872	0.0699	0.3172
10^{-10} M	−0.2714	0.3721	0.0503	0.3217
10^{-9} M	−0.3060	0.3234	0.0087	0.3147
10^{-8} M	−0.3372	0.2931	−0.0220	0.3151
10^{-7} M	−0.3450	0.2608	−0.0421	0.3029
10^{-6} M	−0.3723	0.2018	−0.0853	0.2870
10^{-5} M	−0.3768	0.2149	−0.0809	0.2959

MW CLARI				
Concentrations	Sensor 1	Sensor 2	Average	Standard Deviation
10^{-15} M	0.2356	1.2696	0.7526	0.5170
10^{-14} M	0.5043	3.0169	1.7606	1.2563
10^{-13} M	0.7534	5.6357	3.1946	2.4411
10^{-12} M	0.9321	8.8369	4.8845	3.9524
10^{-11} M	1.1118	12.6809	6.8964	5.7846
10^{-10} M	1.3347	17.8506	9.5926	8.2579
10^{-9} M	1.5415	23.7623	12.6519	11.1104
10^{-8} M	1.7610	30.4630	16.1120	14.3510
10^{-7} M	1.9612	36.7914	19.3763	17.4151
10^{-6} M	2.1190	47.1884	24.6537	22.5347
10^{-5} M	2.3429	53.0738	27.7083	25.3654

MW ERIT				
Concentrations	Sensor 1	Sensor 2	Average	Standard Deviation
10^{-15} M	−0.015635615	0.038991603	0.0117	0.0273
10^{-14} M	−0.100054729	0.022152197	−0.0390	0.0611
10^{-13} M	−0.175895981	−0.049136708	−0.1125	0.0634
10^{-12} M	−0.21358818	−0.035794482	−0.1247	0.0889
10^{-11} M	−0.236529308	−0.021220127	−0.1289	0.1077
10^{-10} M	−0.271723587	−0.064155612	−0.1679	0.1038
10^{-9} M	−0.281178095	−0.076722196	−0.1790	0.1022
10^{-8} M	−0.323372253	−0.097834268	−0.2106	0.1128
10^{-7} M	−0.341931737	−0.157576559	−0.2498	0.0922
10^{-6} M	−0.365188325	−0.144436119	−0.2548	0.1104
10^{-5} M	−0.391553519	−0.178290771	−0.2849	0.1066

RW AZI				
Concentrations	Sensor 1	Sensor 2	Average	Standard Deviation
10^{-15} M	−0.2076	−0.1596	−0.1836	0.0240
10^{-14} M	−0.2727	−0.2153	−0.2440	0.0287
10^{-13} M	−0.3113	−0.2844	−0.2979	0.0134
10^{-12} M	−0.3346	−0.3097	−0.3222	0.0124
10^{-11} M	−0.3527	−0.3283	−0.3405	0.0122
10^{-10} M	−0.3658	−0.3463	−0.3561	0.0097
10^{-9} M	−0.3869	−0.3613	−0.3741	0.0128

RW CLARI				
Concentrations	Sensor 1	Sensor 2	Average	Standard Deviation
10^{-15} M	0.0270	−0.1053	−0.0392	0.0662
10^{-14} M	−0.0222	−0.1742	−0.0982	0.0760
10^{-13} M	−0.0678	−0.2171	−0.1425	0.0746
10^{-12} M	−0.1209	−0.2542	−0.1876	0.0666
10^{-11} M	−0.1415	−0.2771	−0.2093	0.0678
10^{-10} M	−0.2003	−0.2979	−0.2491	0.0488
10^{-9} M	−0.2159	−0.3188	−0.2674	0.0514

RW ERIT				
Concentrations	Sensor 1	Sensor 2	Average	Standard Deviation
10^{-15} M	−0.0106	−0.0199	−0.0152	0.0046
10^{-14} M	−0.0168	−0.0306	−0.0237	0.0069
10^{-13} M	−0.0196	−0.0365	−0.0280	0.0084
10^{-12} M	−0.0268	−0.0429	−0.0348	0.0080
10^{-11} M	−0.0310	−0.0629	−0.0469	0.0160
10^{-10} M	−0.0347	−0.0593	−0.0470	0.0123
10^{-9} M	−0.0381	−0.0601	−0.0491	0.0110

10^{-8} M	−0.3907	−0.3726	−0.3817	0.0090
10^{-7} M	−0.4047	−0.3869	−0.3958	0.0089
10^{-6} M	−0.4097	−0.4065	−0.4081	0.0016
10^{-5} M	−0.4167	−0.4264	−0.4215	0.0049

10^{-8} M	−0.2211	−0.3363	−0.2787	0.0576
10^{-7} M	−0.2422	−0.3592	−0.3007	0.0585
10^{-6} M	−0.2646	−0.3770	−0.3208	0.0562
10^{-5} M	−0.2740	−0.3916	−0.3328	0.0588

10^{-8} M	−0.0397	−0.0644	−0.0521	0.0123
10^{-7} M	−0.0429	−0.0735	−0.0582	0.0153
10^{-6} M	−0.0416	−0.0725	−0.0571	0.0155
10^{-5} M	−0.0446	−0.0778	−0.0612	0.0166

Table S4. Normalized Impedance data (Ω) reproducibility of the ZnO based sensors devices produced with 50% O_2, when immersed in MW and RW at different AZI, CLA and ERY concentrations.

MW AZI				
Concentrations	Sensor 1	Sensor 2	Average	Standard Deviation
10^{-15} M	0.0117	−0.0077	0.0020	0.0097
10^{-14} M	0.0276	−0.0084	0.0096	0.0180
10^{-13} M	0.0443	−0.0083	0.0180	0.0263
10^{-12} M	0.0555	−0.0086	0.0235	0.0320
10^{-11} M	0.0671	−0.0071	0.0300	0.0371
10^{10} M	0.0789	−0.0055	0.0367	0.0422
10^{-9} M	0.0873	−0.0037	0.0418	0.0455
10^{-8} M	0.0985	−0.0005	0.0490	0.0495
10^{-7} M	0.1100	−0.0001	0.0550	0.0551
10^{-6} M	0.1195	0.0023	0.0609	0.0586
10^{-5} M	0.1287	0.0066	0.0677	0.0611

MW CLARI				
Concentrations	Sensor 1	Sensor 2	Average	Standard Deviation
10^{-15} M	0.0108	0.0040	0.0074	0.0034
10^{-14} M	0.0277	0.0126	0.0201	0.0075
10^{-13} M	0.0412	0.0224	0.0318	0.0094
10^{-12} M	0.0531	0.0304	0.0418	0.0113
10^{-11} M	0.0629	0.0392	0.0510	0.0118
10^{-10} M	0.0750	0.0480	0.0615	0.0135
10^{-9} M	0.0864	0.0558	0.0711	0.0153
10^{-8} M	0.0965	0.0633	0.0799	0.0166
10^{-7} M	0.1068	0.0765	0.0917	0.0151
10^{-6} M	0.1179	0.0839	0.1009	0.0170
10^{-5} M	0.1278	0.0911	0.1094	0.0183

MW ERIT				
Concentrations	Sensor 1	Sensor 2	Average	Standard Deviation
10^{-15} M	0.0418	0.0171	0.0295	0.0123
10^{-14} M	0.1028	0.0544	0.0786	0.0242
10^{-13} M	0.1482	0.0806	0.1144	0.0338
10^{-12} M	0.1872	0.1059	0.1465	0.0407
10^{-11} M	0.2282	0.1266	0.1774	0.0508
10^{-10} M	0.2638	0.1464	0.2051	0.0587
10^{-9} M	0.2978	0.1631	0.2304	0.0674
10^{-8} M	0.3347	0.1857	0.2602	0.0745
10^{-7} M	0.3654	0.1979	0.2816	0.0837
10^{-6} M	0.4034	0.2153	0.3094	0.0940
10^{-5} M	0.4290	0.2257	0.3273	0.1017

RW AZI				
Concentrations	Sensor 1	Sensor 2	Average	Standard Deviation
10^{-15} M	0.0396	0.0213	0.0305	0.0091
10^{-14} M	0.0248	0.0282	0.0265	0.0017
10^{-13} M	0.0114	0.0275	0.0194	0.0081

RW CLARI				
Concentrations	Sensor 1	Sensor 2	Average	Standard Deviation
10^{-15} M	0.0067	0.0014	0.0040	0.0026
10^{-14} M	0.0380	0.0035	0.0207	0.0172
10^{-13} M	0.0701	0.0059	0.0380	0.0321

RW ERIT				
Concentrations	Sensor 1	Sensor 2	Average	Standard Deviation
10^{-15} M	0.0365	0.0107	0.0236	0.0129
10^{-14} M	0.0520	0.0191	0.0355	0.0165
10^{-13} M	0.0598	0.0215	0.0407	0.0192

MW AZI				
10^{-12} M	0.0123	0.0274	0.0199	0.0075
10^{-11} M	0.0154	0.0296	0.0225	0.0071
10^{-10} M	0.0163	0.0310	0.0237	0.0073
10^{-9} M	0.0176	0.0330	0.0253	0.0077
10^{-8} M	0.0196	0.0343	0.0269	0.0073
10^{-7} M	0.0253	0.0358	0.0306	0.0053
10^{-6} M	0.0241	0.0371	0.0306	0.0065
10^{-5} M	0.0256	0.0390	0.0323	0.0067

MW CLARI				
10^{-12} M	0.1441	0.0104	0.0773	0.0668
10^{-11} M	0.1876	0.0131	0.1003	0.0873
10^{-10} M	0.2149	0.0176	0.1163	0.0987
10^{-9} M	0.2387	0.0215	0.1301	0.1086
10^{-8} M	0.2630	0.0302	0.1466	0.1164
10^{-7} M	0.2751	0.0341	0.1546	0.1205
10^{-6} M	0.2878	0.0375	0.1626	0.1252
10^{-5} M	0.3007	0.0404	0.1706	0.1302

MW ERIT				
10^{-12} M	0.0642	0.0243	0.0443	0.0200
10^{-11} M	0.0685	0.0254	0.0470	0.0216
10^{-10} M	0.0729	0.0283	0.0506	0.0223
10^{-9} M	0.0775	0.0273	0.0524	0.0251
10^{-8} M	0.0810	0.0279	0.0544	0.0265
10^{-7} M	0.0847	0.0329	0.0588	0.0259
10^{-6} M	0.0892	0.0422	0.0657	0.0235
10^{-5} M	0.0907	0.0377	0.0642	0.0265

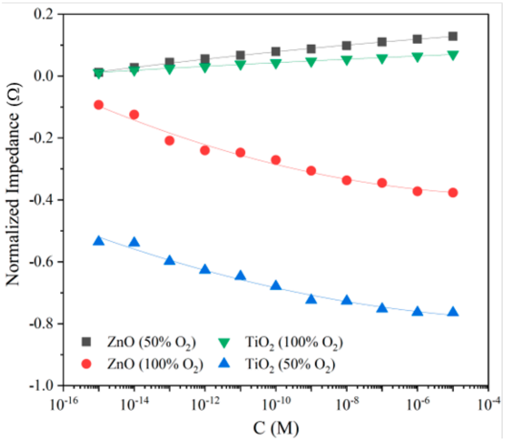

Figure S1. Normalized impedance as a function of azithromycin concentration in MW at fixed frequencies: 1, 1.58, 2.51 and 2.51 × 10^3 Hz measured by the TiO_2 (100% O_2) sensor, TiO_2 (50% O_2) sensor, ZnO (100% O_2) sensor, and ZnO (50% O_2) sensor, respectively. To note that the percentage inside the parentheses correspond to the oxygen percentage used to reactive sputtered the corresponding metallic target.

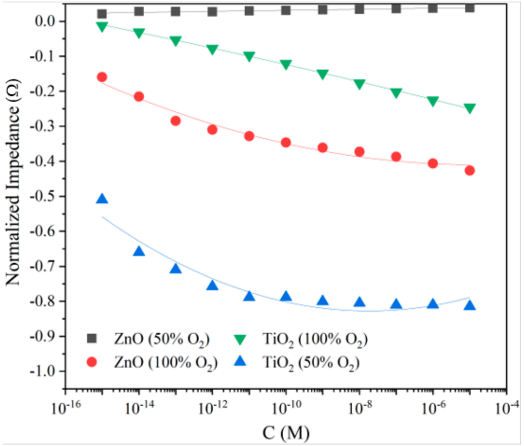

Figure S2. Normalized impedance as a function of azithromycin concentration in RW at fixed frequencies: 1, 1.58, 3.98 × 10^3 and 6.31 × 10^4 Hz measured by TiO_2 (50% O_2) sensor, ZnO (100% O_2) sensor, TiO_2 (100% O_2) sensor, and ZnO (50% O_2) sensor, respectively. To note that the percentage inside the parentheses correspond to the oxygen percentage used to reactive sputtered the corresponding metallic target.

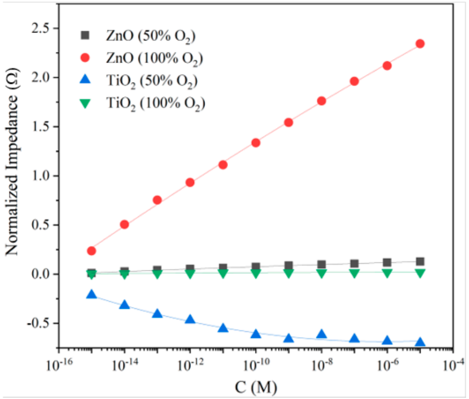

Figure S3. Normalized impedance as a function of clarithromycin concentration in MW at fixed frequencies: 1, 1, 2.51, 2.51 Hz measured by the TiO_2 (50% O_2) sensor, TiO_2 (100% O_2) sensor, ZnO (50% O_2) sensor and ZnO (100% O_2) sensor, respectively. To note that the percentage inside the parentheses correspond to the oxygen percentage used to reactive sputtered the corresponding metallic target.

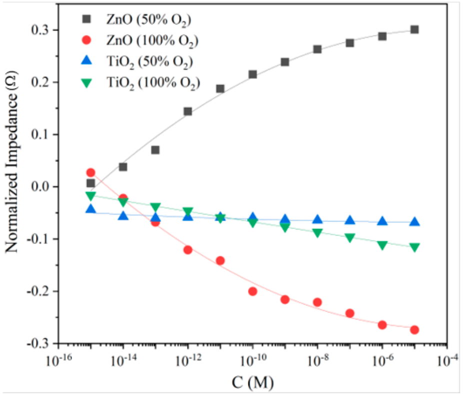

Figure S4. Normalized impedance as a function of clarithromycin concentration in RW at fixed frequencies: 1, 3.98 × 10^2, 2.51 × 10^3 and 10^6 Hz measured by the ZnO (100% O_2) sensor, ZnO (50% O_2) sensor, TiO_2 (100% O_2) sensor, and TiO_2 (50% O_2) sensor, respectively. To note that the percentage inside the parentheses correspond to the oxygen percentage used to reactive sputtered the corresponding metallic target.

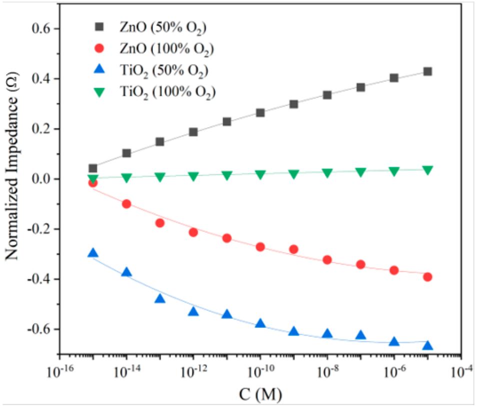

Figure S5. Normalized impedance as a function of erythromycin concentration in MW at fixed frequencies: 1, 1.58 × 10^2 and 2.51 × 10^3 Hz measured by the TiO_2 (50% O_2) sensor, ZnO (50% O_2) sensor, ZnO (100% O_2), TiO_2 (100% O_2) sensor, respectively. To note that the percentage inside the parentheses correspond to the oxygen percentage used to reactive sputtered the corresponding metallic target.

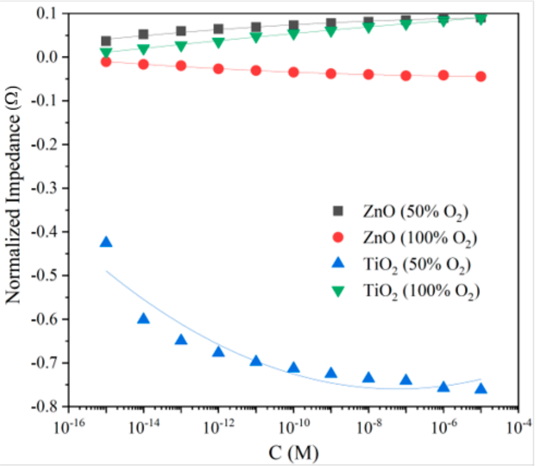

Figure S6. Normalized impedance as a function of erythromycin concentration in RW at fixed frequencies: 1, 10, 2.51 × 10^4 and 10^6 Hz measured by the TiO_2 (50% O_2), TiO_2 (100% O_2) sensor, ZnO (50% O_2) sensor, and ZnO (100% O_2) sensor, respectively. To note that the percentage inside the parentheses correspond to the oxygen percentage used to reactive sputtered the corresponding metallic target.

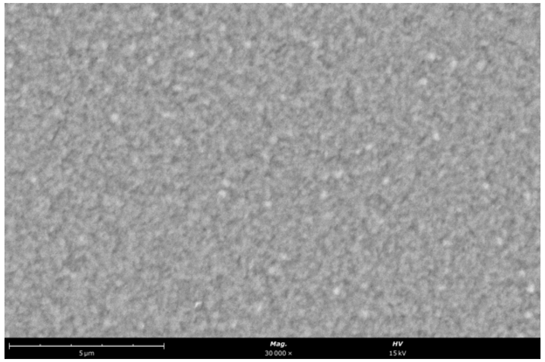

(**a**)ZnO film deposited with 50% O_2

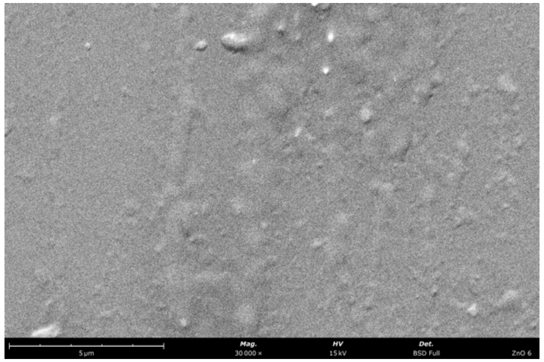

(**b**) ZnO film deposited with 100% O_2

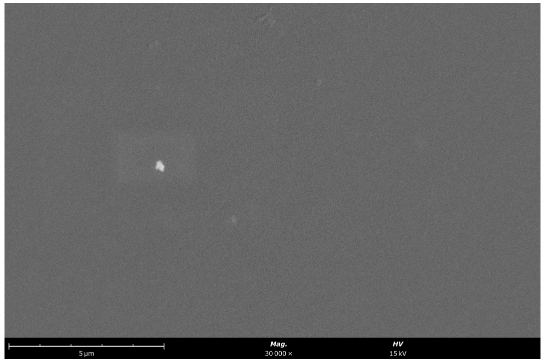

(**c**) TiO_2 film deposited with 50% O_2

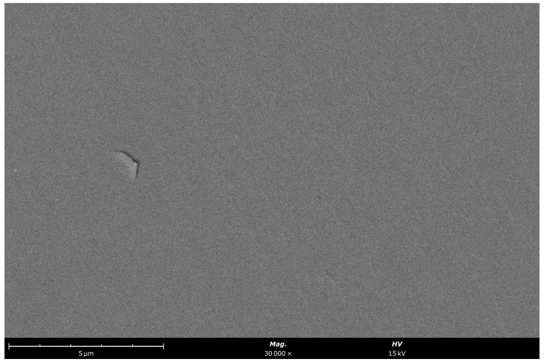

(**d**) TiO_2 film deposited with 100% O_2

Figure S7. SEM images with 30,000 times magnification for (**a**) ZnO 50%; (**b**) ZnO 100%; (**c**) TiO_2 50%; (**d**) TiO_2 100% (scale bar size: 5 µm).
